# Development of ocular dominance columns across rodents and other species: revisiting the concept of critical period plasticity

**DOI:** 10.3389/fncir.2024.1402700

**Published:** 2024-07-05

**Authors:** Toru Takahata

**Affiliations:** Systems Neuroscience Section, Center for the Evolutionary Origins of Human Behavior, Kyoto University, Kyoto, Japan

**Keywords:** ocular dominance columns, albino, critical period plasticity, immediate-early gene, geniculo-cortical inputs

## Abstract

The existence of cortical columns, regarded as computational units underlying both lower and higher-order information processing, has long been associated with highly evolved brains, and previous studies suggested their absence in rodents. However, recent discoveries have unveiled the presence of ocular dominance columns (ODCs) in the primary visual cortex (V1) of Long-Evans rats. These domains exhibit continuity from layer 2 through layer 6, confirming their identity as genuine ODCs. Notably, ODCs are also observed in Brown Norway rats, a strain closely related to wild rats, suggesting the physiological relevance of ODCs in natural survival contexts, although they are lacking in albino rats. This discovery has enabled researchers to explore the development and plasticity of cortical columns using a multidisciplinary approach, leveraging studies involving hundreds of individuals—an endeavor challenging in carnivore and primate species. Notably, developmental trajectories differ depending on the aspect under examination: while the distribution of geniculo-cortical afferent terminals indicates matured ODCs even before eye-opening, consistent with prevailing theories in carnivore/primate studies, examination of cortical neuron spiking activities reveals immature ODCs until postnatal day 35, suggesting delayed maturation of functional synapses which is dependent on visual experience. This developmental gap might be recognized as ‘critical period’ for ocular dominance plasticity in previous studies. In this article, I summarize cross-species differences in ODCs and geniculo-cortical network, followed by a discussion on the development, plasticity, and evolutionary significance of rat ODCs. I discuss classical and recent studies on critical period plasticity in the venue where critical period plasticity might be a component of experience-dependent development. Consequently, this series of studies prompts a paradigm shift in our understanding of species conservation of cortical columns and the nature of plasticity during the classical critical period.

## Introduction

The primate cerebral cortex comprises layers and columns, with a six-layered structure widely conserved across mammalian species. While cortical columns have been traditionally associated with highly-evolved species like primates and carnivores, they were believed to be absent in rodents (Hubel and Wiesel, 1977; Mountcastle, 1997; Krubitzer, 2007; Kaschube et al., 2010; Kaas, 2012). Although some researchers have considered the rodent barrel cortex as a form of cortical column (Meyer et al., 2013), it should be more accurately described as a topographic map of the whisker somatosensory system with distinct septa. Consequently, cortical columns are commonly viewed as computational units within intelligent brains.

Ocular dominance columns (ODCs) in the striate cortex (V1) serve as exemplary cortical columns, where neurons predominantly respond to either the right or left eye (Hubel and Wiesel, 1977). These columns have been extensively studied in macaques, displaying beautifully organized stripes approximately 400 μm wide (Horton and Hocking, 1998). Near the peripheral monocular crescent, ocular dominance tilts towards the contralateral eye, with ODCs appearing as small patches for the ipsilateral eye. While ODCs are most distinct in layer 4, they exhibit continuity from layer 2 to layer 6 (Takahata et al., 2009).

The functional significance of ocular dominance columns (ODCs) was initially presumed to be associated with stereoscopic three-dimensional (3-D) vision (Hubel and Wiesel, 1977). The segregation of pathways from the two eyes at early stages of the visual pathway, including the lateral geniculate nucleus (LGN) and V1, was thought to facilitate binocular disparity coding necessary for stereoscopic vision. However, this assumption was challenged by the observation that some primate species, such as squirrel monkeys and marmosets, lack ODCs (Spatz, 1989; Horton and Adams, 2005).

Nevertheless, recent discoveries have revealed the presence of ODCs in pigmented rats (Laing et al., 2015), prompting a reevaluation of the role and significance of ODCs. In this context, I provide an overview of the history of ODC research, the characteristics of rat ODCs, and their developmental traits. Rat ODCs develop in a manner dependent on visual experience, with monocular deprivation causing a significant shift in ocular dominance towards the intact fellow eye (Olavarria et al., 2021; Zhou et al., 2023). Patchy domains resembling ODCs have also been observed in the dorsal lateral geniculate nucleus (dLGN), although they appear to be more resilient to abnormal visual experiences (Li et al., 2023).

Furthermore, I discuss cross-species differences in the geniculo-cortical network and the functional implications of ODCs, considering their role in visual processing and the potential insights they offer into the organization of the visual system across different species. At the end, I hypothesize that plasticity during the classical critical period may be better understood within the framework of experience-dependent developmental processes. By recognizing it, we gain insights into the conserved mechanisms of cortical development across species and the nature of plasticity during critical periods. This series of studies highlights better understanding of the interplay between experience, development, and plasticity in shaping the functional organization of the brain.

### Methods to reveal ODCs

**FIGURE 1:**
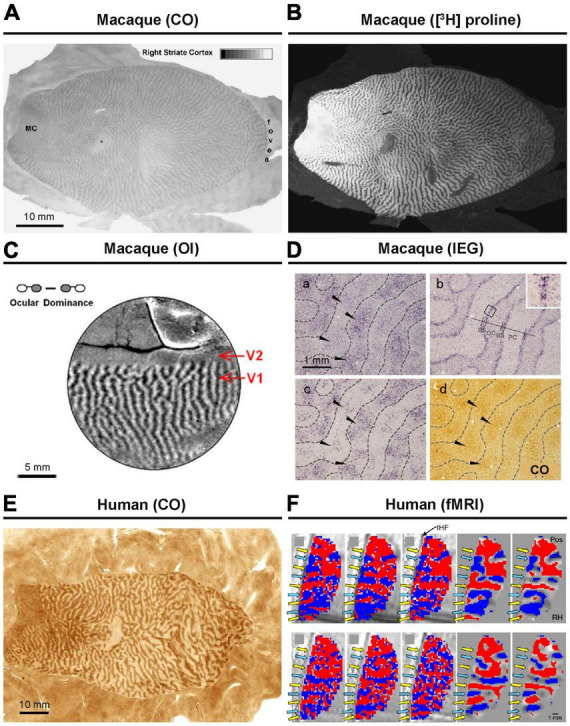
Ocular dominance columns (ODCs) revealed by various methods. In macaques, ODCs has been revealed by CO histochemistry **(A)**, transneuronal anterograde tracing **(B)**, intrinsic signal optical imaging **(C)**, and immediate-early gene (IEG) expression **(D)**. Further details can be found in the original research papers (Horton and Hocking, 1998; Takahata et al., 2009; Lu et al., 2010). In human, **(E)** CO histochemistry **(F)** and fMRI have been applied to reveal ODCs (Adams et al., 2007; Yacoub et al., 2007).

The presence of ODCs was initially suggested through sequential recordings of electrophysiology in cats and macaques (Hubel and Wiesel, 1977). Subsequently, the organization and representation of ODCs were further elucidated using histological techniques, including monocular injection of radioactive, transsynaptic anterograde tracers, and cytochrome oxidase (CO) staining following monocular inactivation (Figures 1A, B; Horton and Hocking, 1998). While CO histochemistry was initially believed to reveal the general activity of cortical neurons (Wong-Riley, 1989), it was later understood to primarily reflect the activity of thalamo-cortical afferent terminals, as evidenced by its staining pattern’s similarity to that of VGLUT2, an endogenous marker protein for thalamocortical afferent terminals, or cortical dendrites that directly receive geniculo-cortical inputs (Takahata, 2016; Yao et al., 2021). Consequently, both transsynaptic anterograde tracers and CO histochemistry essentially reveal the same substance and do not visualize the ocular dominance activity pattern of cortical neurons beyond geniculo-striate recipient domains/layers.

In the late 1980s, the uptake of radio-labeled 2-deoxy-D-glucose (2-DG) was utilized to map neuronal activity differences in deprived and nondeprived ODCs (Tootell et al., 1988). More recently, researchers have employed live imaging techniques such as functional MRI, multiphoton calcium imaging, and intrinsic signal optical imaging to visualize cortical maps (Figure 1C), revealing not only ODCs but also their spatial relationships with other functional features of V1, such as orientation columns (Lu and Roe, 2008; Garg et al., 2019; de Hollander et al., 2021). However, these methods often require substantial pre-treatment, have limited resolution, and/or only capture a small portion of V1.

One of the efficient methods for revealing ODCs involves examining the expression patterns of neuronal activity-dependent genes. Transcriptions of genes such as c-Fos, Egr-1 (Zif268), and Arc are triggered by intracellular calcium and cAMP, which are enhanced through the activation of NMDA receptors, L-type calcium channels, and/or trkB receptors (Rajadhyaksha et al., 1999; Bramham et al., 2008). It is thought that the expression of these genes is enhanced by neuronal activity where action potentials coincide with synaptic activity, earning them the designation of “immediate-early genes (IEGs).” The demonstration of ODCs through IEG expression after monocular inactivation was first introduced in macaques by Chaudhuri (1997). It was capable of revealing functional ocular compartments in all layers throughout V1 that have not been revealed by CO histochemistry (Figure 1D; Takahata et al., 2009). Therefore, examining IEG is considered the most sensitive currently available method for revealing ODCs, although it is important to note that different methods demonstrate different aspects of ODCs.

### ODCs in primates and carnivores

ODCs have been extensively studied in macaques, where they manifest as regularly organized stripes with a width of approximately 400 μm (Horton and Hocking, 1996c,^1998^). In the macaque visual cortex, the representation of the optic disk (approximately 15° in eccentricity near the horizontal meridian) is ipsilaterally dominant, while the most peripheral visual field (usually > 40° in eccentricity) is contralaterally dominant. Thus, these two portions of V1 lack ODCs. ODC representation is patchy for the ipsilateral eye just outside the peripheral monocular visual field, typically beyond 20° in eccentricity.

**FIGURE 2:**
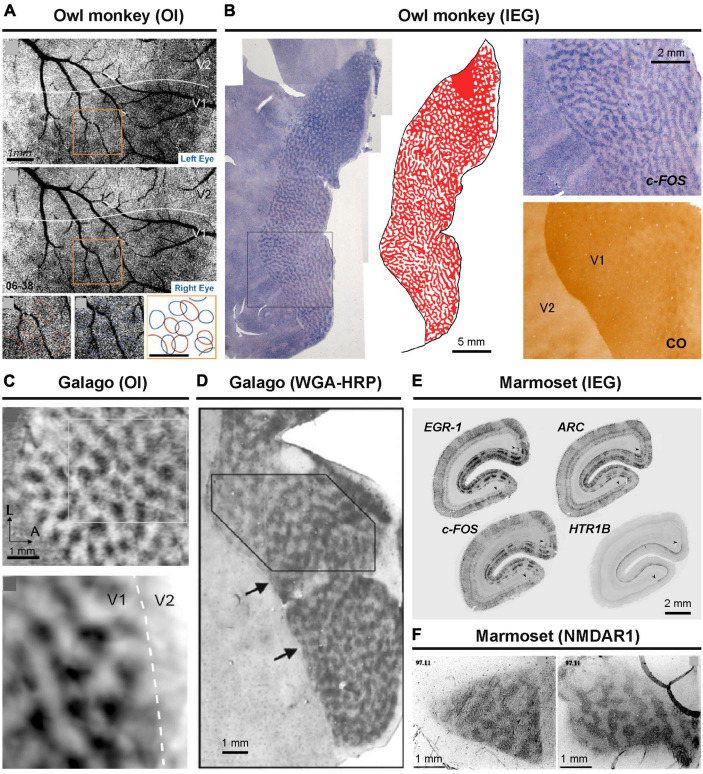
ODCs revealed in New World and prosimian primates. In owl monkeys, optical imaging **(A)** and IEG expression **(B)** have been applied (Kaskan et al., 2007; Takahata et al., 2014). In prosimian galagos, optical imaging **(C)** and anterograde tracing of WGA-HRP **(D)** have been applied to reveal ODCs (Xu et al., 2005; Olavarria et al., 2022). In marmosets, IEG expression **(E)** and NMDAR1 expression **(F)** have revealed ODCs (Chappert-Piquemal et al., 2001; Nakagami et al., 2013).

The presence of ODCs has been confirmed in chimpanzees and humans as well (Figures 1E, F; Tigges and Tigges, 1979; Horton and Hedley-Whyte, 1984; Cheng et al., 2001; de Hollander et al., 2021). Extensive studies on ODCs have also been conducted in cats and ferrets using various methodologies (Crair et al., 1997; Issa et al., 1999; Kasamatsu and Imamura, 2020). Among other primates, ODCs have been observed in spider monkeys, Capuchin monkeys, talapoin monkeys, green monkeys, and vervet monkeys mostly using traditional transsynaptic tracers or the CO method (Guillery et al., 1984; Florence et al., 1986; Hess and Edwards, 1987; Florence and Kaas, 1992; Chaudhuri et al., 1995). These classical comparative studies are listed in Table 1 of a paper by Horton and Hocking (1996b). Previously, owl monkeys were thought to lack ODCs based on tracer and CO methods (Kaas et al., 1976; Livingstone, 1996), but more recent research employing intrinsic optical imaging and IEG expression has confirmed their presence (Figures 2A, B; Kaskan et al., 2007; Takahata et al., 2014). Interestingly, ODCs were even found to extend into V2 in owl monkeys. The presence of ODCs was also questioned in prosimian galagos previously, but they have been clearly revealed by optical imaging and tracer methods in recent studies (Figures 2C, D; Xu et al., 2005; Olavarria et al., 2022).

The prevailing theory regarding marmosets suggests that they possess ODCs only during their juvenile stage, gradually losing them as the visual system matures (Florence et al., 1986). However, the evidence supporting this theory does not appear very strong. While it has been widely described in a number of review articles and textbooks, only one classical paper (Spatz, 1989) has demonstrated it, and that study used a small number of marmosets at each age group. They employed the traditional method of radioactive transsynaptic tracer to reveal ODCs but only examined them in coronal sections, without analyzing tangential sections or specifying the portion of V1 under investigation. More recently, the presence of ODCs has been suggested by optical imaging and confirmed by activity-dependent gene expression in adult marmosets (Figures 2E, F; Chappert-Piquemal et al., 2001; Roe et al., 2005; Nakagami et al., 2013). As a result, the theory of developmental loss of ODCs is not frequently mentioned in recent discussions.

**FIGURE 3:**
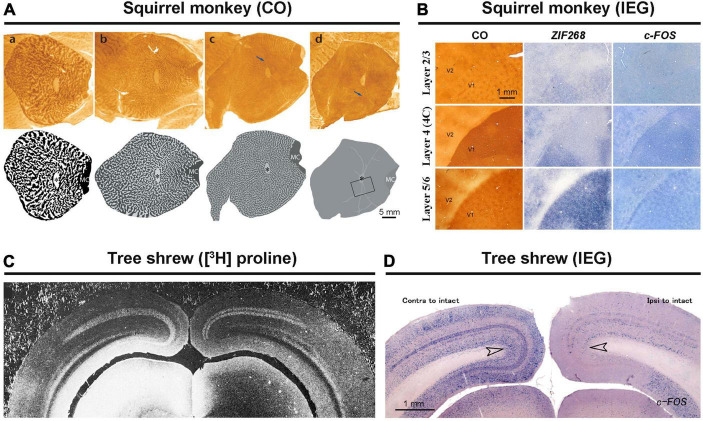
Exceptional species regarding ODCs. In squirrel monkeys, capricious expression of ODCs was revealed by CO staining **(A)** (Adams and Horton, 2003). The attempt to reveal ODCs using IEG expression resulted in failure **(B)** (Li et al., 2021). Both transneuronal tracers **(C)** and IEG expressions **(D)** have shown that ocular dominance is segregated into layers but not into columns in tree shrews (Hubel and Wiesel, 1977; Takahata and Kaas, 2017).

Squirrel monkeys present a controversial case regarding ODCs. While CO histochemistry and transneuronal tracing have revealed ODCs in some individuals of squirrel monkeys, they are absent in others. In some instances, ODCs are observed in one portion of V1 but not in the rest (Figure 3A; Horton and Hocking, 1996b; Adams and Horton, 2003). Despite examining squirrel monkeys using IEG expression, none exhibited ODC-like staining patterns in their V1s (Figure 3B; Li et al., 2021). Perhaps, squirrel monkeys form ODCs depending on genetic polymorphism or enriched environment as observed in cats (Kaschube et al., 2003). An electrophysiological study showed that neurons in the visual cortex of a squirrel monkey are capable of disparity coding (Livingstone et al., 1995), which posed a strong counterargument against the theory that ODCs are necessary for stereoscopic 3-D vision (Adams and Horton, 2009). However, this was done in only one individual, and ODC presence was not evaluated in that monkey. After all, ODCs in squirrel monkeys are still controversial.

Tree shrews present another challenging case of ocular dominance arrangement. While they are segregated into layers in V1, they lack columns (Figure 3C; Humphrey et al., 1977). This segregation pattern has been demonstrated through electrophysiology, transneuronal tracer studies, and examination of IEG expression (Figure 3D; Fitzpatrick and Raczkowski, 1990; Takahata and Kaas, 2017; Cang et al., 2023). This unique ocular segregation has not been reported in any other species.

### Discovery of ODCs in Long-Evans rats

**FIGURE 4:**
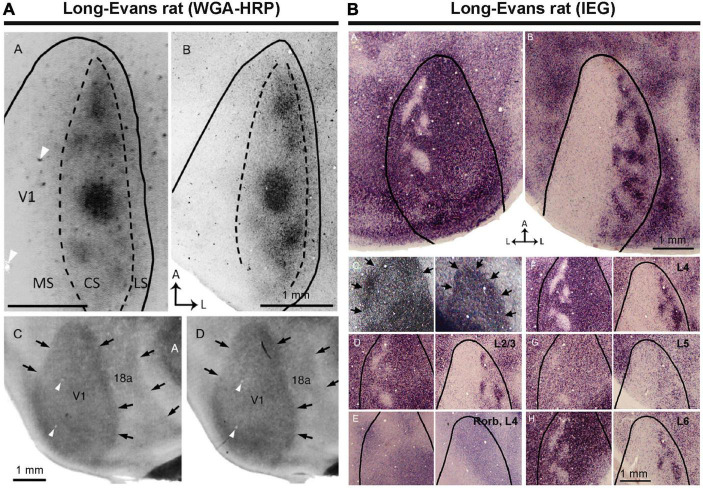
ODCs of rats were first identified by transneuronal tracer **(A)**, and IEG expressions **(B)** (Laing et al., 2015).

**FIGURE 5:**
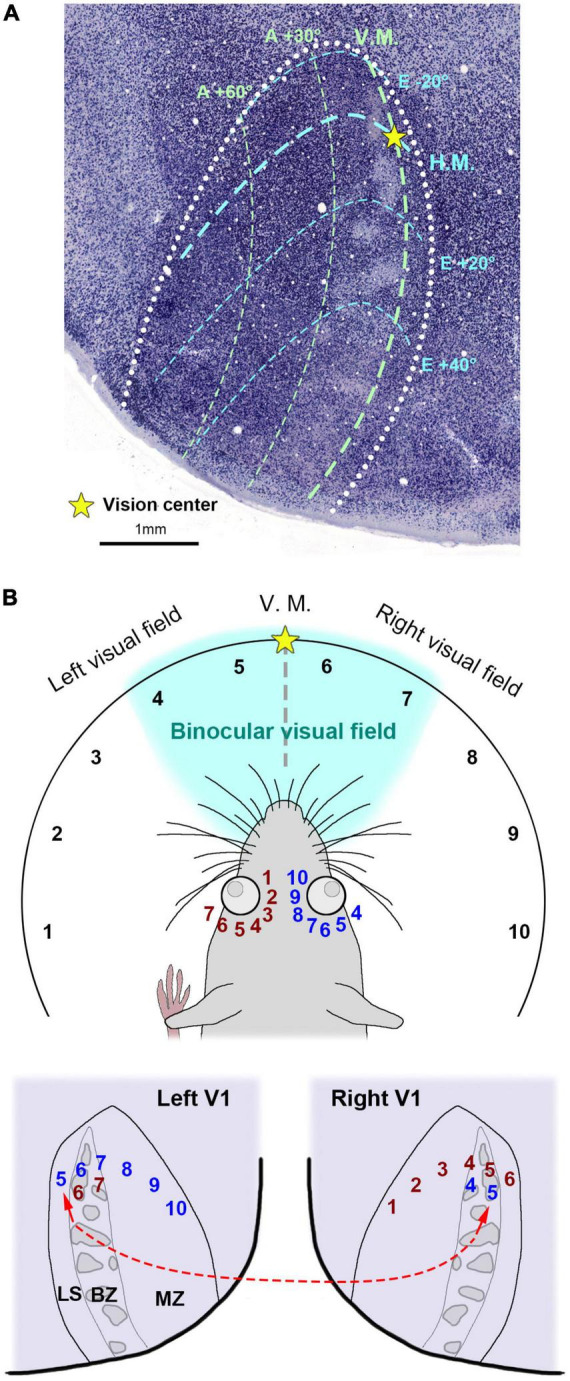
**(A)** The retinotopic map of the rat V1 related to ODCs inferred from previous studies (Drager, 1975; Wagor et al., 1980; Murakami et al., 2022). The V1 border is drawn by white dots. Right is lateral and upper is anterior. H.M./horizontal meridian; V.M./vertical meridian; A/azimuth degrees; E/elevation degrees. **(B)** An illustration of the rat visual field and its hypothetical relationship to the V1 retinotopic maps. Each visual field is represented by numerical labels, with corresponding representations shown in the retina and V1. Briefly, visual fields 5-10 are represented in the left V1 via the right eye, while visual fields 6 and 7 are represented in the binocular zone (BZ) of the left V1 via the left eye. Notably, visual field 5 is represented in the lateral segment (LS) of the left V1, despite being in the ipsilateral (left) visual field. Similarly, visual field 6 is also represented in the LS of the right V1. The left V1 LS is interconnected with the right V1 BZ as shown in the red broken line. MZ/monocular zone.

There were previous attempts to uncover ODCs in rats and mice, but earlier studies concluded that rodents lack ODCs (Drager, 1974; Hubel and Wiesel, 1977). Subsequently, no further attempts were made for over four decades. However, one study demonstrated the presence of ODCs in Long-Evans rats (Laing et al., 2015). They utilized the tracer WGA-HRP and observed patchy callosal projections from V1 innervating the contralateral side of V1 in Long-Evans rats (Figure 4A). Since callosal projection typically corresponds with ODCs in other species, they suspected Long-Evans rats might possess patchy ODCs as well. Injecting WGA-HRP into one eye, they observed patchy labeling in the binocular zones of Long-Evans V1. Electrophysiology and IEG expression also subsequently demonstrated clear patchy patterns in the binocular zones of V1 of Long-Evans rats (Figure 4B). As the patchy pattern persisted from layer 2 to layer 6, with patches exhibiting almost symmetric shape and size between hemispheres, they concluded that they were indeed ODCs in rats, marking the first discovery of cortical column structure in rodents. Matched to previously studied retinotopic maps of rodent visual cortex (Drager and Olsen, 1980; Marshel et al., 2011; Ji et al., 2015), ODCs are evident near the central visual field and slightly upper visual field of V1 (Figure 5A).

Intriguingly, ODCs are absent near the lateral edge of V1 (lateral segment; LS), and the tracer data and the IEG expression both suggest that the LS receives exclusive inputs from the contralateral eye. While V1 of primates receives inputs exclusively from the contralateral visual hemifield, it has been suggested that rodent V1 receives inputs from some of the ipsilateral visual field across the vertical meridian, as well as the entire contralateral visual hemifield (Drager and Olsen, 1980; Marshel et al., 2011). Thus, the fact that the LS exclusively receives inputs from the contralateral eye implicates that the LS represents the ipsilateral visual field of the contralateral eye (Figure 5B). Perhaps, some visual field near the vision center is represented in both V1s (visual fields 5 and 6 in Figure 5B). As mentioned earlier, the LS receives massive callosal projections from the ODCs in the other V1 (shown in the red broken line in Figure 5B) (Laing et al., 2015), suggesting that the right and left visual hemifields are connected at the LS in rodents. These anatomical features related to the LS have not been demonstrated until ODCs were revealed in rats.

### Albinos lack ODCs

**FIGURE 6:**
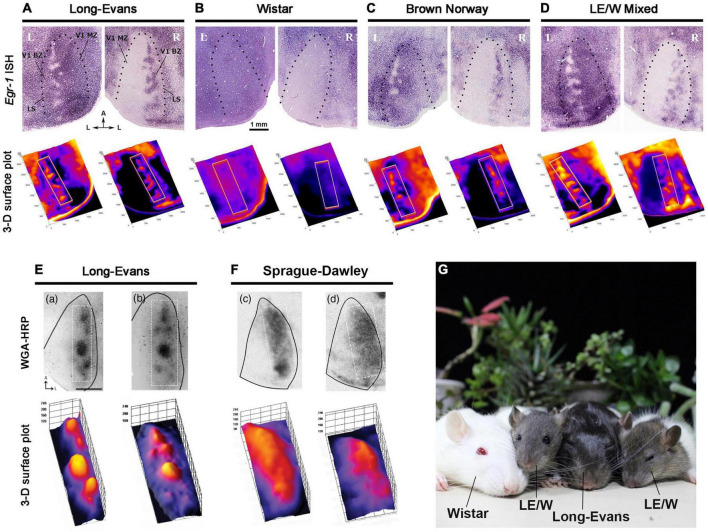
Strain difference of ODC expressions revealed by IEG expressions **(A–D)** (Zhou et al., 2023), and WGA-HRP **(E,F)** (Andelin et al., 2020). ODCs are present in pigmented Long-Evans **(A,E)**, Brown Norway **(C)**, and the first generation of Long-Evans and Wistar (LE/W) **(D)**, but not in albino rats **(B,F)**. **(G)** A family photograph of Long-Evans father, Wistar mother and LE/W litters.

Subsequent studies revealed significant differences between Long-Evans and albino rats. Sprague-Dawley (SD) or Wistar rats did not exhibit any patchy ipsilateral input patterns at all (Figure 6; Andelin et al., 2020; Zhou et al., 2023). Previous studies indicated that the amount of ipsilateral visual inputs in albino mice is only about 30% of that in pigmented mice (Drager and Olsen, 1980). The etiology of this anatomical abnormality remains unknown, although recent research suggests that delayed cell-cycle control in the ciliary margin zone of the retina due to miscommunication between retinal progenitor cells and retinal epithelial cells lacking melanin may play a role (Mason and Slavi, 2020; Slavi et al., 2023). This disruption in the topography of axon guidance molecules like Ephrins and Slits could lead to misrouting of ipsilateral retino-geniculate inputs. Additionally, the retinotopic representation of V1 neurons does not correspond between the two eyes in albino rats, likely due to abnormal topography of retino-geniculate and geniculo-cortical afferents (Rebsam et al., 2012). It seems reasonable to suggest that ODCs do not develop when there is little or no competition between the two eyes in the cortical field. Besides rats, abnormalities of eye-specific domains have been reported in albino green monkeys, white tigers, and Siamese cats as well (Guillery and Kaas, 1973; Kaas and Guillery, 1973; Guillery et al., 1984).

The next question was whether ODC formation is specific to the Long-Evans strain or common among pigmented rats. To address this, Brown Norway rats, the strain closest to wild rats, was examined by IEG expression (Figure 6C; Zhou et al., 2023). As a result, clear patches of ODCs were observed in the binocular zones of their V1, although they were slightly less pronounced compared to those in Long-Evans rats, and patches were sometimes interconnected with each other. This observation is important because it suggests that rats commonly have ODCs in their natural environment, and the presence of ODCs may confer survival benefits.

ODCs were also observed in LE/W mixed rats, the first-generation offspring of Long-Evans fathers and Wistar mothers (Figure 6D; Zhou et al., 2023). This suggests that the deficiency in the albino visual system can be fully compensated for by heterozygous hybrids with Long-Evans rats, even though the hair color of LE/W mixed rats is sometimes brighter than that of pure Long-Evans rats.

### ODCs in the mouse

**FIGURE 7:**
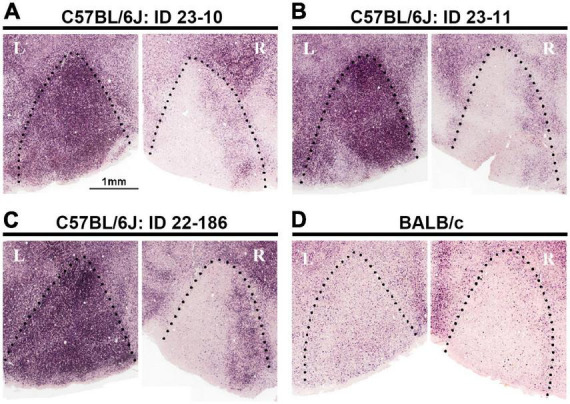
Attempts to reveal ODCs in mice using IEG expression did not show evident columns, but some minor clusters in the binocular zones of V1 in C57BL/6J strain **(A–C)**. In albino BALB/c strain, the IEG expression is low overall, and even the distinction between the monocular and binocular zones is unclear **(D)** (Zhou et al., 2023).

Considering evolutionary traits and the usefulness of mice as a research subject, the possible presence of ODCs in the mouse V1 became the next point of interest. ODCs were investigated through IEG expression in pigmented C57BL/6J mice (Zhou et al., 2023). Similar to pigmented rats, monocular zones exhibited clear uniform inactivation in the contralateral V1 and uniform activation in the ipsilateral V1 to the enucleated eye. In the binocular zones, the activation level was moderate, and some patch-like patterns were moderately observed, although they were not as obvious as in pigmented rats (Figures 7A–C). When albino BALB/c mice were examined, IEG expression was relatively low overall in V1, and even boundaries between the monocular and binocular zones were not as distinct as in albino rats (Figure 7D).

While not distinctly clear enough to be classified as ‘ODCs’, minor clusters of ocular dominance neurons in the C57BL/6J mouse V1 were observed. These clusters may represent prototypes of cortical columns in rodents. It is possible that under more specific conditions, such as an enriched environment, strabismus, or increased thalamo-cortical input activity, these clusters could manifest as more apparent ODCs. Indeed, the formation of ODCs was observed in Ten-m3 mutant mice, where ipsilateral input is increased, although they are not in physiological conditions (Merlin et al., 2013). By investigating which conditions facilitate the formation of ODCs in the mouse V1, we can deepen our understanding of the development and evolution of cortical columns.

**FIGURE 8:**
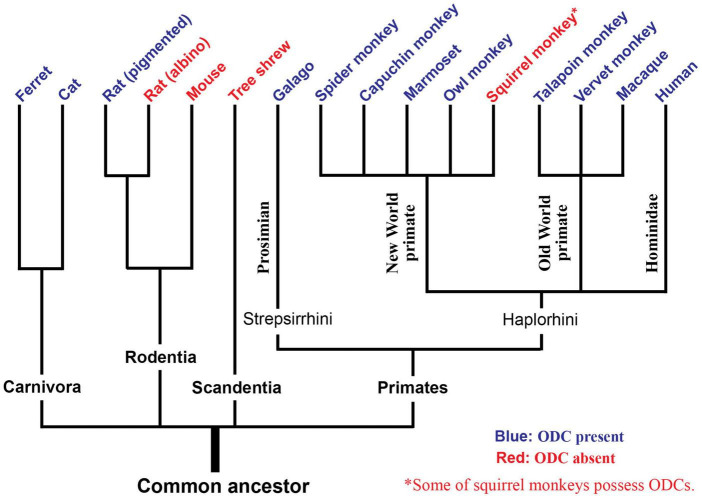
An evolutionary tree regarding the presence of ODCs. Species with blue letters have been shown to possess ODCs, while species with red letters do not. Tree shrews exhibit layer separation of ocular dominance. Some individuals of squirrel monkeys possess ODCs.

The updated phylogenetic tree regarding the presence or absence of ODCs highlights that ODCs are a universal cortical structure across mammalian species rather than being restricted in species closer to humans, with the exceptions of squirrel monkeys and tree shrews (Figure 8).

### Development of rat ODCs

**FIGURE 9:**
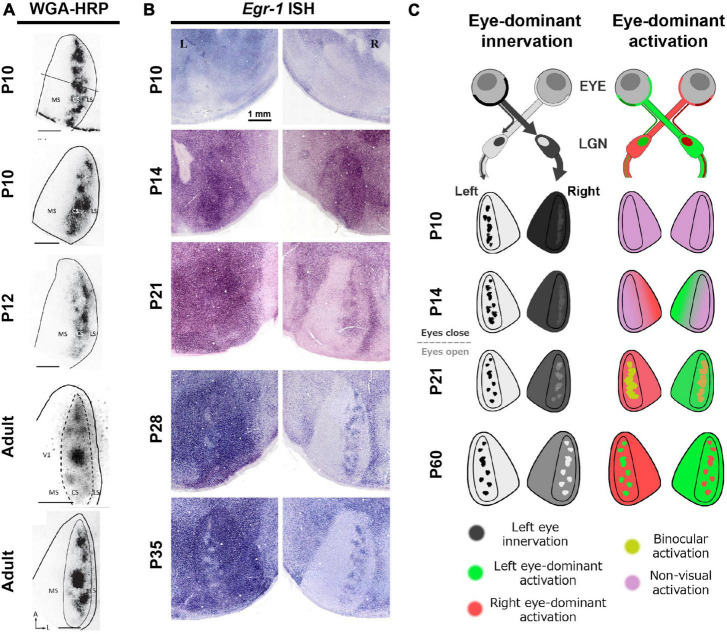
Distinct development of anatomical and physiological ODCs. **(A,B)** While anatomical ODCs revealed by WGA-HRP are already visible before eye-opening **(A)**, physiological ODCs revealed by IEG expression take until 35 days old **(B)** (Olavarria et al., 2021; Zhou et al., 2023). **(C)** A schematic of distinct development of anatomical (left) and physiological (right) ODCs.

Intriguingly, investigations into the development of rat ODCs using different methodologies have yielded contrasting conclusions. A transneuronal tracing study indicated the presence of patchy ipsilateral geniculo-cortical inputs even before the eyes open (<P14) (Figure 9A; Olavarria et al., 2021), consistent with previous findings in non-human primates and carnivores (Horton and Hocking, 1996a; Crair et al., 2001; Katz and Crowley, 2002). However, when ODC development was examined through IEG expression, the ODC pattern did not appear to mature by P35 (Figure 9B) (Zhou et al., 2023). Specifically, most neurons in the binocular zones exhibited high level of IEG expression even after ipsilateral eye-enucleation at around P21 (Figure 9B, P21 left), demonstrating their reactivity to the contralateral eye. On the other hand, ipsilateral patches are visible in the other hemisphere (Figure 9B, P21 right), showing that some neurons in the binocular zones are responsive to the ipsilateral eye. At this stage, many neurons would be reactive to both eyes, but not exclusive to either eye.

This discrepancy depending on methodology suggests that while anatomical ODCs, as indicated by distribution patterns of geniculo-cortical afferents, develop early, physiological ODCs, as indicated by response patterns of cortical neurons, take a much longer period to mature. The reason of this gap can be interpreted as follows (Figure 9C): In rats under 20 days old, even though the geniculo-cortical afferents conveying ipsilateral eye inputs exhibit a patchy distribution in the binocular zones, those conveying contralateral eye inputs are distributed evenly throughout V1, including both the monocular and the binocular zones. This is manifested by uniform dark labeling of WGA-HRP in the contralateral V1 to the injected eye, and by uniform intense expression of IEG in the ipsilateral V1 to the enucleated eye (Figures 9A, B; Olavarria et al., 2021; Zhou et al., 2023). Over the subsequent weeks, some of the contralateral innervation is withdrawn, leading to neurons in the binocular zones becoming responsive to either eye. Recent studies suggest that the orientation selectivity in binocular neurons in layer 2/3 of V1 undergoes binocular alignment through visual experience during this period (Wang et al., 2010; Chang et al., 2020).

Given the observations that the level of IEG expression remains high overall even following monocular enucleation at around eye-opening (Figure 9B, P14), the spiking activity of neurons in V1 is presumably noisy at this stage. Note that cortical IEG expression significantly decreases in cases of cortical TTX or muscimol injection, showing that the IEG expression is dependent on neuronal activity even at this age (Zhou et al., 2023). Perhaps, spontaneous activity of thalamic and/or cortical neurons may be high without sensory inputs as implicated by several studies (Maffei et al., 2004; Siegel et al., 2012; Hagihara et al., 2015), or V1 neurons may be responsive to other sensory modalities as well (Van Brussel et al., 2011; Frangeul et al., 2016; Gribizis et al., 2019). As described below, responses of V1 neurons become tuned and sharpened to one of the eyes through normal visual experiences by P35. The mixture of these components may lead to distinct conclusions depending on which aspect of ODC is observed.

Consistent with the observations in rats above, it has also been reported in primates and carnivores that ODC borders appear to be more clear-cut in the ipsilateral V1 to the eye that is injected transneuronal tracer compared with that in the contralateral V1 (LeVay et al., 1978, 1980; Horton and Hocking, 1996a), suggesting that the contralateral-eye afferents are more widely distributed than the ipsilateral-eye afferents. Additionally, ODCs were not clearly revealed by EGR-1 (ZIF268) expression for the first 6 months after birth in Cebus monkeys, while they are clear in CO histochemistry, suggesting that the maturation of physiological ODCs takes longer than that of anatomical ODCs (Silveira et al., 1996).

### Critical period of ocular dominance plasticity

**FIGURE 10:**
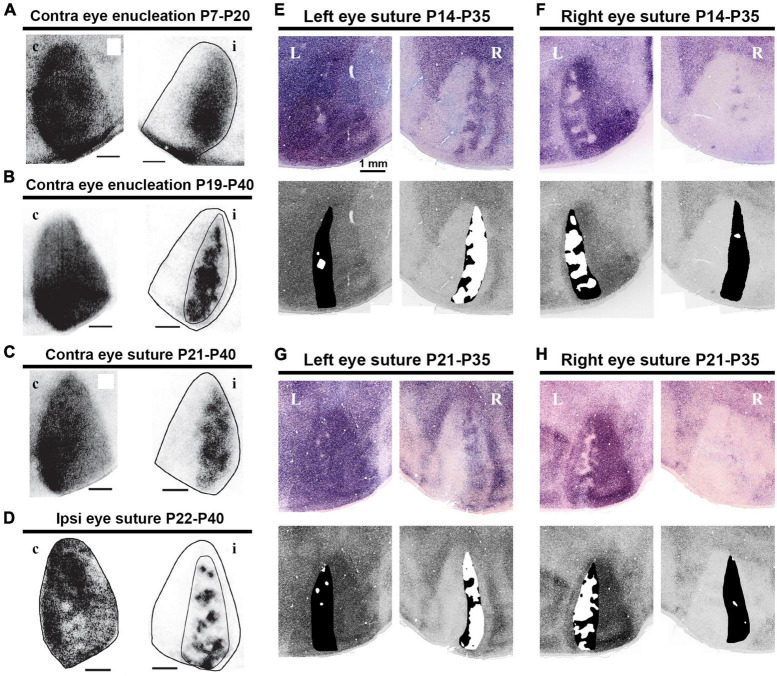
Distinct activity dependence of the development of anatomical and physiological ODCs. **(A–D)** ODC patterns revealed by WGA-HRP in rats raised under various conditions as indicated (Olavarria et al., 2021). c/contralateral to the WGA-HRP injected eye; i/ipsilateral to the WGA-HRP injected eye. **(E–H)** ODC patterns revealed by IEG expressions in rats raised under various conditions as indicated (Zhou et al., 2023). L/left hemisphere; R/right hemisphere. Note that monocular enucleation and monocular eyelid suture shifted ODC sizes in favor of the intact eye in all cases.

The critical period of ocular dominance plasticity is estimated to be between P21 and P35 in both mice and rats (Gordon and Stryker, 1996; Guire et al., 1999; Hensch, 2004; Medini, 2011). Consistent with this, studies have shown that the size of rat ODCs is significantly influenced by abnormal visual experience during this period in favor of the non-deprived eye (Figure 10) (Olavarria et al., 2021; Zhou et al., 2023). Monocular deprivation shifts the territories of ODCs towards the open eye compared to the normally reared rats. Dark-rearing treatment prevents the maturation of ODCs from occurring after P21 (Zhou et al., 2023).

Previous rodent studies have been primarily conducted using electrophysiological techniques such as recording for single units, multi units, patch clamp, and local field potentials (Gordon and Stryker, 1996; Guire et al., 1999; Hensch, 2004; Medini, 2011). One series of studies utilized IEG expression to estimate ocular dominance shift in mice (Tagawa et al., 2005). The developmental stages suggested by these studies are as follows: (1) the ratio of right/left eye dominance is predetermined even before eye-opening at P14; (2) there is a preparatory period for the critical period, referred to as the “pre-critical period”; (3) mechanisms of the critical period are activated at around P21, allowing for environmental adaptation; (4) the mechanisms of the critical period turn off at P35, and the circuit is consolidated.

**FIGURE 11:**
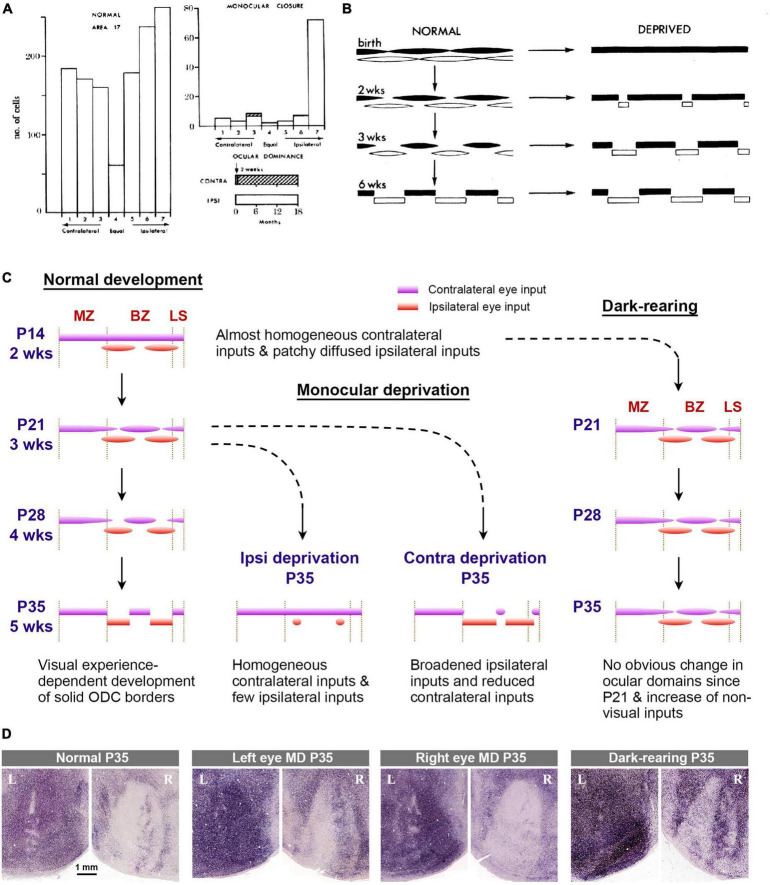
**(A)** Ocular dominance histograms assessed by electrophysiology in normal and monocularly deprived (from 2 weeks to 18 months) macaque monkeys in classical studies [copied from Hubel et al. (1977)]. **(B)** Classical scheme that explains the development and effects of eye closures on ODCs in macaques [copied from Hubel et al. (1977)]. **(C)** Scheme of development and effects of abnormal visual experiences on ODCs in rats. **(D)** ODCs revealed by IEG expression in rats raised in various visual conditions (Zhou et al., 2023). They are consistent with conditions in the rat scheme above.

In primates and carnivores, ocular dominance shift has also been evaluated through electrophysiology (Figure 11A; Olson and Freeman, 1975; Hubel et al., 1977; Issa et al., 1999; Kasamatsu and Imamura, 2020). These classical studies primarily aimed to determine the shift in ipsi/contra response ratio of cortical neurons resulting from monocular deprivation, often with a limited number of subjects. Later, the critical period has also been estimated by assessing the size of ODCs through anatomical evaluations using transneuronal tracers and CO histochemistry to determine the critical period of ocular dominance plasticity (Figure 11B; Hubel et al., 1977; Horton and Hocking, 1997; Chiu and Weliky, 2002; Katz and Crowley, 2002; Keil et al., 2010; Takahata et al., 2018). These studies suggest that ODCs are already formed before birth, and ODCs are most plastic immediately after birth, indicating that there is no “pre-critical period” for primates and carnivores and their ODCs become solidified by 2 to 3 months after birth.

Taken together, there appears to be a significant gap between rodents and primates/carnivores in terms of our understanding of ocular dominance development. However, it should be noted that rodent studies primarily relied on physiological methods to evaluate critical period plasticity, while primate/carnivore studies more relied on anatomical methods. The discovery of rat ODCs has enabled researchers to investigate both anatomical and physiological features of development within the same species, utilizing hundreds of individuals in each study.

Rat ODC studies suggest that the formation of anatomical ODCs precedes physiological ODCs. Their developmental steps seem more gradual than previous theory. They can be outlined as follows (Figures 11C, D): (1) Uniform innervation of geniculo-cortical afferents conveying contralateral eye input & patchy innervation of geniculo-cortical afferents conveying ipsilateral eye input (before eye opening at P14); (2) maturation of synapses and binocular competition (P14 to P21); (3) more maturation of synapses through patterned vision & withdrawal of geniculo-cortical afferents conveying contralateral eye input (P21 to P35).

Overall, the development of rat ODCs appears to incorporate features of both previously suggested ocular dominance development in rodents and primates/carnivores, thereby bridging studies across different species. Despite appearing silent during the ‘pre-critical period’ in mice, the extension of geniculo-cortical fibers and synapse formation/selection likely continues during this period. Similarly, although anatomical ODCs may appear to be already completed before birth in primates/carnivores, cortical neuron responses are likely immature and noisy without clear borders of ODCs neonatally. Consequently, the differences between species are not as distinct as previously estimated.

### Ocular domains in the rat dLGN

**FIGURE 12:**
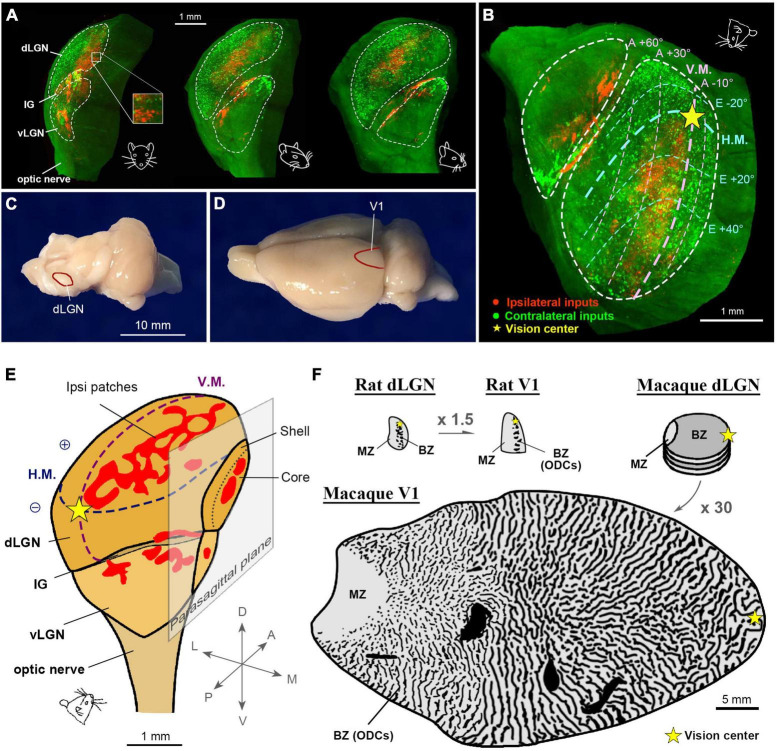
Ocular domains in the pigmented rat LGN. **(A)** Three-dimensional topography of the rat LGN. The contralateral and ipsilateral eye domains are marked with green (CTB-488) and red (CTB-555) tracers, respectively. The rat face indicates the angle of the LGN. **(B)** A specific angle view that exhibits the retinotopic map of the dLGN (Li et al., 2023). **(C,D)** Photographs of a rat brain at a macro scale to exhibit that the sizes of dLGN **(C)** and V1 **(D)** are comparable. **(E)** A three-dimensional illustration of the rat LGN with maps of retinotopy and ocular domains. **(F)** Comparisons of the gross size of the rat dLGN, rat V1, macaque dLGN, and macaque V1 in the same scale. Note that the V1/dLGN ratio is approximately 1.5 in the rat, while it is approximately 30 in the macaque.

While ocular inputs are segregated into layers in early visual pathways of the dorsal lateral geniculate nucleus (dLGN) in primates and carnivores (Kaas et al., 1972; Horton and Hocking, 1996c), it has been believed that ocular inputs are segregated only at the cellular level in the binocular zones of the dLGN in rodents (Hubel and Wiesel, 1977; Espinosa and Stryker, 2012). Studies in Long-Evans rats have suggested that the ipsilateral retino-geniculate domains of the Long-Evans rats are segregated into several islands, and the functional segregation was assumed to be similar to that in primates (Discenza and Reinagel, 2012); however, three-dimensional evaluation using tissue clearing techniques have revealed distinct differences in the topography of the rat dLGN compared to primate counterparts (Figure 12A; Li et al., 2023). At a specific angle, the retinotopic map and ocular domains in the rat dLGN exhibit similarities to those observed in V1 (Figure 12B), facilitating a comparison of the map sizes between the dLGN and V1. At a gross scale, their sizes appear comparable (Figures 12C, D). The patchy ipsilateral domains in the rat dLGN demonstrate a mesh-like organization at any angle (Figure 12E). Comparisons of the sizes of retinotopic map reveal that the V1/dLGN ratio is approximately 1.5 in rats, whereas it is approximately 30 in macaques (Figure 12F), highlighting a substantial species difference in cortical expansion. This difference likely enabled the cortex of monkeys to enlarge the geniculo-cortical afferent sorting filter to accommodate multiple organized columnar domains to a much greater extent than in rodents (Kremkow and Alonso, 2018).

**FIGURE 13:**
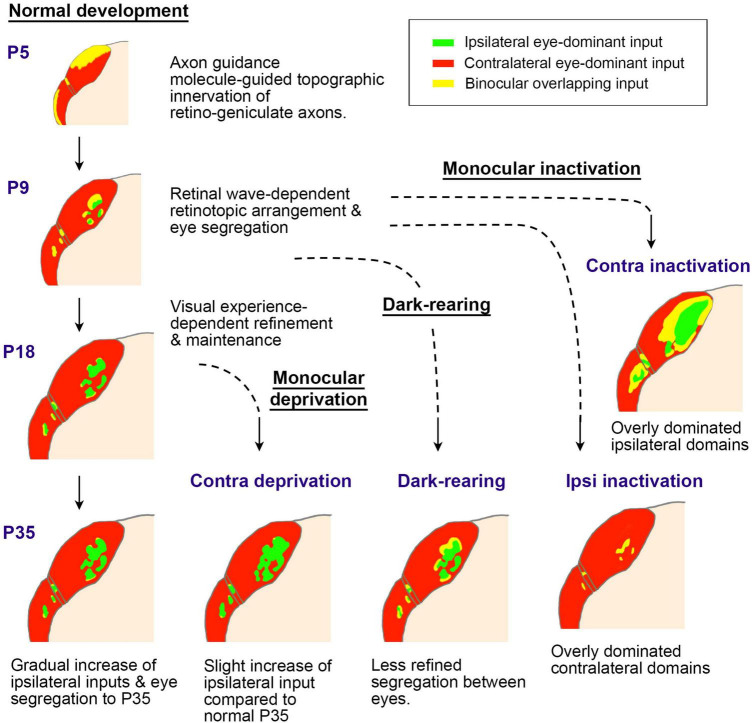
A schematic of postnatal LGN development in rodents regarding ocular domains. Initially, axon guidance molecules roughly guide the topography of innervation patterns (P5). Then, the retinotopic and ocular arrangement is refined through retinal waves (P9). After eye-opening at P14, the LGN continues to grow, with ipsilateral domains slightly enlarging. When the retinal wave is deprived due to eye enucleation or intravitreous TTX injection at around P9, ocular segregation is strongly disrupted. After eye-opening, ocular deprivation by eyelid-suture, dark-rearing, or eye enucleation only shows minor influence on the morphology of the LGN, although the binocular tuning of geniculo-cortical axons is disrupted (Kerschensteiner and Guido, 2017; Liang and Chen, 2020; Olavarria et al., 2021; Tan et al., 2021; Stacy and Van Hooser, 2022; Li et al., 2023).

Nevertheless, the ipsilateral patches of the rat dLGN can be a good model for investigating the critical period for ocular dominance (Figure 13). In primates and carnivores, monocular deprivation treatment results in the shrinkage of LGN neurons (Movshon and Dursteler, 1977; Stacy and Van Hooser, 2022; Duffy et al., 2023) and down-regulation of the expression of many genes and proteins (Higo et al., 2000; Takahata et al., 2010), but the sizes of eye-specific layers do not significantly alter. Additionally, partial retinal lesion at an early postnatal day induces microglia aggregation, but the size of the lesion projection zone of the dLGN appears unaffected (Takahata et al., 2018).

In rodents, it has been elucidated that the topographical inputs of retino-geniculate axons are guided by intrinsic axon guidance molecules, such as Ephrins (Pfeiffenberger et al., 2005). While the segregation of eye territories in the rodent LGN is largely mediated by neuronal activity, it is contingent upon retinal wave activity before eye-opening rather than patterned vision (Assali et al., 2014; Failor et al., 2015). Notably, the dLGN exhibits minimal structural influence from visual experience, while monocular inactivation treatments like monocular enucleation or intravitreous TTX injection at P7-P10 almost completely disrupt eye segregation (Failor et al., 2015; Stacy and Van Hooser, 2022). Thus, the impact of monocular eyelid suture and dark-rearing on the ipsilateral patches of the rat dLGN appears minor, although there is a slightly significant influence on their size (Li et al., 2023). Monocular enucleation at P7-P20 (Figure 10A) exhibits a larger impact on ODC formation even more than monocular enucleation at P19-P40 (Figure 10B; Olavarria et al., 2021), even though it is in the so-called pre-critical period. This may seem counterintuitive, but it can be interpreted as being caused by the disruption of eye domains in the dLGN rather than cortically oriented changes.

A recent calcium imaging study on geniculo-cortical afferents revealed that binocular response and visual tuning properties are significantly and permanently decreased in mice subjected to critical period monocular deprivation compared to normally reared mice, despite comparable architectural features (Huh et al., 2020). This shift in response by monocular deprivation was also observed even in adult mice (Jaepel et al., 2017), suggesting that geniculate responses may be affected at synaptic level rather than the architecture level during the critical period (Dumenieu et al., 2021).

## Discussion

**FIGURE 14:**
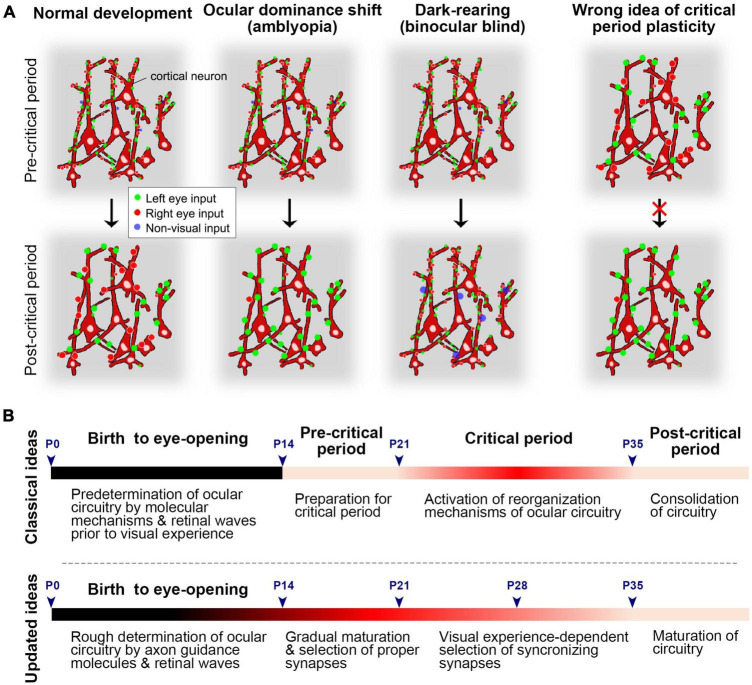
**(A)** Schematics of normal development, ocular dominance shift (amblyopia), and dark-rearing (binocular blind) before and after the critical period at the synaptic level. Synapses of cortical neurons might be dynamic and immature in the pre-critical period. They are selected and matured through visual experiences, with unnecessary synapses pruned out. In the absence of patterned vision, visual synapses do not mature, but non-visual inputs, such as somatosensory inputs, may become dominant in the visual cortex. Previously, it was thought that already matured circuit is given a chance to reorganize during the critical period, but this concept may need updating (on the right). **(B)** The time-course of development regarding ocular dominance in rodents. In the previous theory, the pre-matured ocular dominance circuit is given a chance to reorganize to adopt to the environment during the critical period. However, the circuit is more likely immature and dynamic before and during the critical period, and its development is more gradual than previously estimated.

The ocular dominance system has emerged as a key model for studying neuronal plasticity, leading to the concept of ‘critical period plasticity’ (Hubel et al., 1977). However, the term ‘neuronal plasticity’ refers to the brain’s capacity to reorganize itself by forming new neural connections or synapses throughout life in response to experiences or neuronal damage (Kaas et al., 1983). Previously, it was believed that the ocular dominance circuit is formed via intrinsic mechanisms even before eye-opening (Crowley and Katz, 2002), thus, the bias of ocular preference following monocular deprivation during juvenile period was referred to as ‘ocular dominance plasticity.’ However, studies on rat ODCs suggested that developmental changes, such as synaptic refinement and maturation, continue at least until P35 even in normally reared rats (Zhou et al., 2023). This continuous developmental timeline is consistent with recent studies based on cellular and molecular changes during the critical period (Wang et al., 2010; Espinosa and Stryker, 2012; Hooks and Chen, 2020; Tan et al., 2021). Considering the developmental sequences, the previously defined ‘critical period’ seems to represent a developmental phase where binocular competition plays a crucial role in determining territory allocation (Figure 14).

Below, I outline the remaining questions and hypotheses to be addressed in future studies. First, the reason why thalamo-cortical afferents are sorted in a patchy manner between the two eyes in V1 even before eye-opening remains unknown. The distribution of geniculo-cortical afferents is patchy in V1 between LGN layers even before retino-geniculate afferents reach the LGN (Crowley and Katz, 2000), suggesting that this sorting is independent of retinal activity, such as retinal waves, and likely follows an intrinsic developmental program. One hypothesis is that specific molecular guidance cues are expressed in a patchy manner in V1, attracting thalamo-cortical afferents from either eye. Although some efforts have been made to identify such genes (Tomita et al., 2013), no effective chemoattractants for ODCs have been discovered yet. Another possibility is that chemoattractants or chemorepellents are differentially expressed in LGN layers, attracting same-layer (eye) afferents or repelling different-layer (eye) afferents, thus sorting them within limited spaces. While this kind of molecular study is challenging in primates and carnivores, it can be more feasibly conducted in rodents.

**FIGURE 15:**
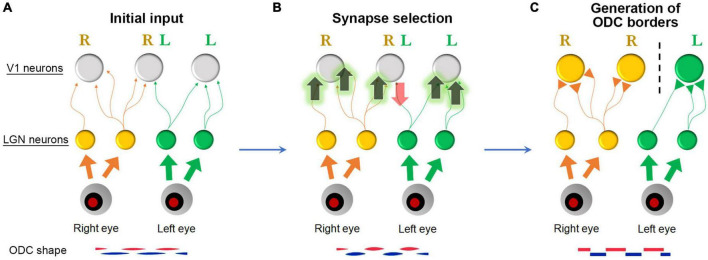
A Schematic of ODC border generation. **(A)** Initially, ocular dominance projections are roughly segregated with many overlapping projections. **(B)** When spiking activity of presynaptic axons is correlated with spiking activity of postsynaptic neurons, these synapses are facilitated. However, when it is not, these synapses are eliminated (Hebbian principle) (Linsker, 1986). **(C)** Consequently, neurons near the border respond strongly and exclusively to either eye, and the column borders become evident.

Second, we need to understand what causes the substantial differences in ODC patterns across species. ODC patterns can range from highly organized stripes to convoluted beads or no discernible pattern at all. One hypothesis suggests that increasing the cortical area per binocular visual point (i.e., the size of the afferent sorting filter) strengthens ocular dominance segregation through a power law function (Najafian et al., 2019). While this model explains the differences well among New World and Old World monkeys, it does not account for the fact that pigmented rats exhibit clear ODCs in their small binocular zones of V1. Therefore, the size of the cortical area and the receptive fields of afferents are not the sole factors contributing to cross-species differences. Another possibility is that the cellular capacity for Hebbian plasticity varies among species. In species with ODCs, postsynaptic V1 neurons or presynaptic geniculo-cortical afferents might respond more robustly to synaptic rearrangement based on action potential correlation, as shown in Figure 15, compared to species without ODCs. This could be due to higher levels of molecules related to plasticity, such as neurotrophic factors, vesicular neurotransmitters, extracellular matrix proteins, and activity-dependent transcription factors (Harris et al., 1997; Pizzorusso et al., 2002; Melo et al., 2013; Ataman et al., 2016; Hrvatin et al., 2018; Kasamatsu and Imamura, 2020). These hypotheses can be explored, and candidate genes can be identified using advanced genetic screening techniques like scRNA-seq. Because mice have a minor cluster of ocular dominance cells, the function of these candidate molecules can be tested by introducing them into mice to see if the clusters turn into ODCs.

While IEG expression is a useful indicator of the physiological state of cortical neurons, employing more direct physiological methods such as calcium imaging and intrinsic-signal optical imaging would provide a more comprehensive characterization of rat ODCs. It should be tested with live imaging whether the development of physiological ODCs takes until P35, as revealed by IEG expression. In addition, the influence of monocular deprivation should be examined and compared between juvenile and adults in rats to confirm that adults have limited capacity of ocular dominance plasticity. These techniques would not only facilitate the more convincing identification of ODCs and enable comparison with other species, but also allow the exploration of other cortical domains, such as orientation columns and illumination domains, in rodents. Despite previous studies denying the presence of orientation domains even in Long-Evans rats (Ohki et al., 2005), it is possible that these domains were overlooked due to factors like inappropriate visual stimuli or other methodological issues.

Furthermore, calcium imaging would enable the study of critical period plasticity issues mentioned earlier. By employing calcium imaging, the time-lapse changes in ocular dominance of individual neurons, or even individual spines, can be monitored over a period of several weeks (Mizuno et al., 2014; Chang et al., 2020; Tan et al., 2022). Additionally, if axonal GCaMP is expressed in geniculate cells, the responses in geniculo-cortical afferents and cortical neurons can be studied separately (Sadakane et al., 2015). In classical understanding, the ocular dominance of each neuron is thought to be predetermined before eye-opening and remains relatively stable unless the animal experiences abnormal visual conditions, such as monocular deprivation (Crowley and Katz, 2000). Under this framework, ocular dominance shifts during the critical period would be considered “plastic changes”. However, more recent perspectives suggest that neuronal responses are inherently fragile, noisy, and multimodal, with eye dominance continuously fluctuating even when the animal is exposed to a normal environment during the juvenile critical period (Mataga et al., 2004; Wilson et al., 2016; Chen et al., 2022). If this is the case, ocular dominance shifts should be regarded as experience-dependent development rather than mere plasticity. Studying these dynamics would be more feasible in rat ODCs due to the ease of morphological observation compared to single neuron responses. This approach would provide deeper insights into the mechanisms underlying ocular dominance and its plasticity during critical periods.

The last major question is: What are the physiological benefits of ODCs? Previously, the idea that cortical columns like ODCs are merely a byproduct of developmental steps, and have no functional significance has been dominant, mainly because squirrel monkeys lack of ODCs (Horton and Adams, 2005). However, it is surprising that ODCs are present in the small binocular zones of the rat V1, despite their disruption of the retinotopic map. Given that Brown-Norway rats, a strain close to wild rats, also possess ODCs, we many need to reconsider their significant physiological advantages for survival. In primates, the eyes are primarily aligned and moved in tandem, preserving retinotopic relationships. While rats and mice sometimes move their eyes in tandem, they often break vergence and rely more on head movements than saccades when pursuing prey (Wallace et al., 2013; Michaiel et al., 2020). Additionally, rodent eyes are positioned on the sides of their heads, leading to the common belief that they do not utilize binocular vision. However, the presence of ODCs suggests this is not necessarily true.

One hypothesis is that the alignment of orientation preference is adjusted between the two eyes in V1 layer 4, and neurons then use this information for binocular disparity coding in later stages (Wang et al., 2010; Chang et al., 2020). Another possibility is that neurons achieve greater efficiency by clustering (forming columns), which shortens wiring (Mitchison, 1991; Bullmore and Sporns, 2012). Neurons with corresponding responses tend to connect with each other; if they are located nearby, they can save space and energy, making computation more efficient. Testing these hypotheses experimentally would be challenging because disturbing ODCs would likely also disrupt other domains, such as the retinotopic map and orientation domains, and then the effect of ODCs would be blurred. Therefore, a theoretical approach may be more reasonable for studying these questions.

In addition to the ocular dominance system of the visual cortex, the concepts discussed in this article are likely applicable to other nervous systems, including motor and somatosensory systems, as well as higher-order association areas for language and cognitive skills. In summary, the previously defined “critical period” may represent one aspect of a developmental time window during which experience-dependent synaptic selection occurs. Nervous system development is likely more gradual than previously believed, occurring both before and possibly even after the traditionally defined critical period. Cortical columns can form even in rodents when sufficient space and the benefits of neuronal clustering are available.

## Author contributions

TT: Writing – original draft, Visualization, Validation, Resources, Project administration, Methodology, Investigation, Funding acquisition, Formal analysis, Data curation, Conceptualization.
